# Measuring fast stochastic displacements of bio-membranes with dynamic optical displacement spectroscopy

**DOI:** 10.1038/ncomms9162

**Published:** 2015-10-06

**Authors:** C. Monzel, D. Schmidt, C. Kleusch, D. Kirchenbüchler, U. Seifert, A-S Smith, K. Sengupta, R. Merkel

**Affiliations:** 1 Institute of Complex Systems 7 (ICS-7): Biomechanics, Forschungszentrum Jülich GmbH, 52425 Jülich, Germany; 2 Aix-Marseille Université, CNRS, Centre Interdisciplinaire de Nanoscience de Marseille UMR 7325, 13288 Marseille, France; 3 Institut für Theoretische Physik, Department für Physik, and the Excellence Cluster: Engineering of Advanced Materials, Friedrich Alexander Universität Erlangen-Nürnberg, 91058 Erlangen, Germany; 4 II. Institut für Theoretische Physik, Fakultät 8: Mathematik und Physik, Universität Stuttgart, 70550 Stuttgart, Germany; 5 Division of Physical Chemistry, Ruđer Bošković Institute, 10000 Zagreb, Croatia

## Abstract

Stochastic displacements or fluctuations of biological membranes are increasingly recognized as an important aspect of many physiological processes, but hitherto their precise quantification in living cells was limited due to a lack of tools to accurately record them. Here we introduce a novel technique—dynamic optical displacement spectroscopy (DODS), to measure stochastic displacements of membranes with unprecedented combined spatiotemporal resolution of 20 nm and 10 μs. The technique was validated by measuring bending fluctuations of model membranes. DODS was then used to explore the fluctuations in human red blood cells, which showed an ATP-induced enhancement of non-Gaussian behaviour. Plasma membrane fluctuations of human macrophages were quantified to this accuracy for the first time. Stimulation with a cytokine enhanced non-Gaussian contributions to these fluctuations. Simplicity of implementation, and high accuracy make DODS a promising tool for comprehensive understanding of stochastic membrane processes.

## Abstract (web summary)

Precise quantification of stochastic motions of biological membranes is limited by a lack of suitable detection methods. Here Monzel *et al*. develop dynamic optical displacement spectroscopy to measure stochastic membrane displacements at 20 nm/10 μs spatiotemporal resolution.

## Graphical abstract

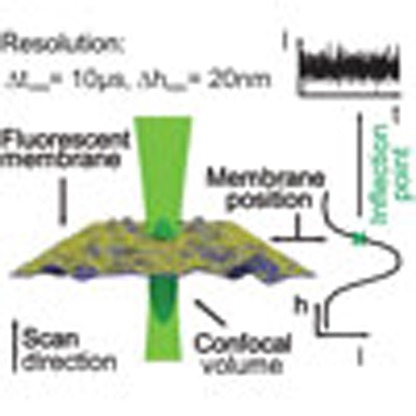

Stochastic remodelling of cellular membranes are essential for many life processes. For example, they play a significant role during cadherin and integrin mediated adhesion^1^,^2^,^3^, they have an influence on substrate sensing and protrusion formation during migration^4^,^5^ and facilitate vesicle budding and curvature induced trafficking processes^6^,^7^. Bending fluctuations of the membrane are an integral component of such remodelling process. In red blood cells (RBCs), such fluctuations are thought to prevent cell–cell adhesion and their modification is a marker of specific diseases^8^,^9^,^10^. In nucleated cells too, modification in fluctuations have been linked to pathology^11^ and recent studies point to a critical role of nuclear envelope fluctuations for chromatin dynamics in *Drosophila* embryos and mouse embryonic stem cells^12^,^13^. The role of fluctuations in stabilizing mitochondria or the endoplasmic reticulum were mooted but are yet to be measured^14^,^15^.

In the past, membrane fluctuations have mostly been quantified on giant unilamellar vesicles^16^,^17^,^18^,^19^,^20^,^21^,^22^ (GUVs) or RBCs^23^,^24^,^25^,^26^, with very few reports on nucleated cells^1^,^3^. The theory of thermally driven bending fluctuations of membranes was developed for both fluid membranes (relevant to GUVs^27^,^28^,^29^) and cytoskeleton scaffolded membranes (relevant for RBCs^23^,^26^). For fluid membranes, the theory is now considered to be well established but experiments at high frequencies and small bending fluctuations are still challenging^22^,^24^,^29^. In case of RBCs, in addition to thermal contribution, an additional active contribution has been suggested both theoretically^26^ and experimentally^25^ but this is still a matter of debate.

In the context of cells, stochastic activity is expected to be inherent to many membrane processes, for example those involving ion pumps^19^. However, due to the lack of suitable experimental tools to accurately measure membrane fluctuations in the complex optical environment of cells, the nature of active fluctuations in cellular membranes and their modulation during the life cycle of a cell is yet to be studied in detail. Several techniques have been developed to measure membrane fluctuations, including flicker spectroscopy^23^, contour analysis^19^, diffraction phase microscopy^25^ and reflection interference contrast microscopy (RICM)^20^,^30^. These techniques often use camera based detection^17^,^18^,^19^,^20^,^21^,^22^,^23^,^25^,^29^ with limited time resolution, and/or rely on refractive index induced contrast^17^,^18^,^19^,^20^,^21^,^22^,^23^,^24^,^25^,^28^,^29^, which is impossible to accurately quantify in nucleated cells due to the presence of organelles and inhomogeneous protein distribution causing ill-defined variations in refractivity. Furthermore, in a given technique, only a specific part of the cell could be accessed—for example, along the equator^23^,^25^ or close to a substrate^1^,^3^,^28^.

A manifold of other technical advancement based on fluorescence correlation spectroscopy (FCS) has been reported during the last decade. These include two-focus or dual-color scanning approaches that provided novel insight into the structural organization of the cell membrane^31^ and enabled quantification of binding affinities *in vivo*^32^, and z-scan FCS^33^, where measurements are recorded at subsequent points along the *z* axis, and which eliminates the need for an extrinsic calibration. Recent developments further combined spectroscopic and super-resolution techniques to reveal the dynamics of transient lipid aggregates at nanometric scales^34^. FCS maps of fast molecular dynamics inside living cells and tissues were realized in a spatially resolved manner using image correlation spectroscopy^35^, spatiotemporal image cross-correlation spectroscopy, k-Space image correlation spectroscopy and raster image correlation spectroscopy^36^ or the combined approaches of single plane illumination–FCS^37^ and stimulated emission depletion–raster image correlation spectroscopy^38^. While these FCS related techniques are highly successful in characterizing the ensemble behaviour of individual molecules or small clusters, these techniques are not suitable to study the motion of huge molecular complexes such as bilayers, whose collective motion and bending fluctuations need to be detected with high precision over rapidly sampled time intervals.

In this work, we introduce a novel methodology called dynamic optical displacement spectroscopy (DODS) which circumvents these issues and can measure membrane fluctuations even in the complex optical environment of nucleated cells. The DODS set-up is based on FCS, which is currently a standard technique to measure molecular diffusion and interaction processes^31^,^39^,^40^,^41^. DODS takes advantage of the fast and sensitive detection inherent to FCS and thus measures membrane fluctuations with 20-nm axial and 10-μs temporal resolution. In addition, as in FCS, DODS measurements can be performed on any chosen part of the cell membrane.

We first validate DODS on model membranes and show that the values of known physical parameters are satisfactorily recovered using standard membrane theory. Next, using DODS to explore the flickering of RBCs, we recover reported results and show that addition of ATP indeed increases the non-Gaussian component but only at the rim. Finally, DODS is applied to a nucleated cell type—human macrophage—to measure hitherto undetectable displacements of the plasma membrane. Quantification of the displacement-histograms and autocorrelation functions shows substantial impact of cytokine stimulation.

## Results

### The principle

To perform DODS, the membrane under study is labelled with a fluorescent dye and placed in the illuminated confocal volume of a standard FCS set-up. In contrast to FCS, where intensity flickers arise from the diffusion of fluorophores, the key idea of DODS is to measure intensity variations originating exclusively from the physical motion of the membrane. This is possible, since (1) the intensity in the confocal volume follows a well-known distribution that can be measured accurately in each experiment and (2) any signals from diffusion processes are easily suppressed, as outlined below. Detected intensity flickers are then converted into membrane fluctuations in a highly controlled manner using an experimentally determined relation.

**Figure 1:**
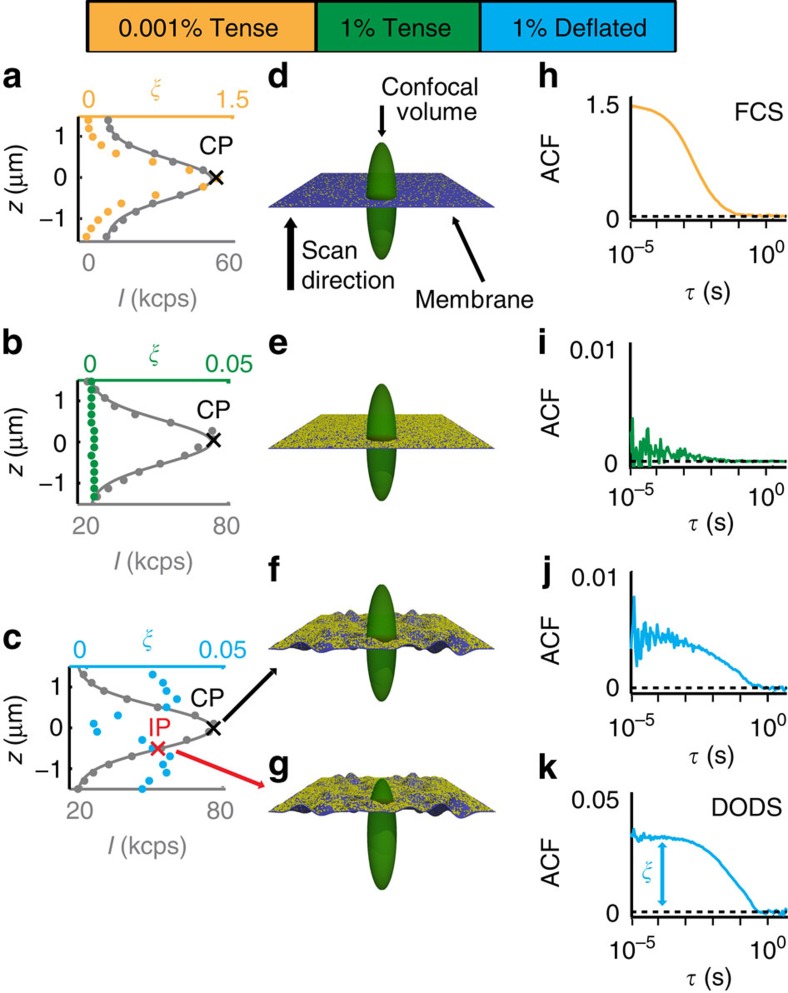
From FCS to DODS. FCS and DODS measurements on the distal membrane of a GUV. (**a**-**c**) *z*-axial scans with measured intensity, *I*, (grey dots) and Gaussian fit (grey line) in kilo counts per second (kcps). *ξ* is the correlation amplitude of the recorded ACF (coloured dots); (**d**–**g**) sketch of the membrane (dark blue sheet) with different fluorophore concentration (yellow dots) in the confocal volume; (**h**–**k**) ACF measured at indicated point (central point, CP, or inflection point, IP). (**a**,**d**,**h**) standard FCS with tense, that is non-fluctuating membrane, low fluorophore concentration (*ξ* exhibits a single peak at CP, and ACF is recorded at the intensity peak CP); (**b**,**e**,**i**) suppression of FCS signal with high fluorophore concentration in tense membrane (*ξ* is low throughout the scan and ACF exhibits a very low signal at CP); (**c**,**f**,**j**) and (**g**,**k**)) DODS with deflated that is fluctuating membrane and high fluorophore concentration (*ξ* has peaks at the IPs of *I*(*z*), the ACF recorded at CP exhibits a low signal (**f**,**j**), while the signal at IP is high (**g**,**k**)).

We demonstrate the principle of DODS using standard FCS as a starting point and GUV membranes as example: In FCS, the membrane is labelled with a fluorescent dye at low concentration (0.001%, see Methods section) and intensity variations *δI*(*t*) result from the diffusion of a very small number of fluorophores in the confocal volume. Illustrated here as a model membrane (Fig. 1a,d,h), the membrane in our experiment is the distal surface of a GUV, which is tensed by immersion in a hypotonic solution. The tension thus generated in the membrane suppresses its bending fluctuations. When performing axial scans of the membrane (Fig. 1a–c), the Gaussian intensity distribution of the illuminating beam, *I*, is recovered and a value of the autocorrelation amplitude, *ξ*, at each axial position is obtained. In case of FCS, *ξ* exhibits a single peak distribution and measurements are performed at the central point of the confocal volume where the intensity signal is highest (Fig. 1h).

If the fluorophore concentration in the membrane is increased to 1%, the FCS signal vanishes and the autocorrelation function (ACF) flattens (Fig. 1b,e,i). The ACF, and consequently *ξ*, remain low at any point of the axial scan (Fig. 1i). In this case dye number fluctuations are so low that all signal originating from fluorophore diffusion is suppressed since it is lost in the noise. As a consequence this data defines the noise level of the system.

Next, a GUV is immersed in a hypertonic solution and is thus rendered floppy. In this situation, its membrane undergoes strong bending fluctuations. Intriguingly, in this case, membrane displacements within the confocal volume with its inhomogeneous intensity distribution, give rise to a significant recovery of intensity flickers (Fig. 1c,f,g,j,k). For a deflated GUV with 1% fluorophore concentration, this signal is free of any diffusion contribution and *δI*(*t*) results exclusively from physical displacements of the membrane. Note that presence of 1% labelled lipids is not expected to impact the fluidity or flexibility of the membrane (for example, compare concentrations for phase transition changes in refs 42, 43). Furthermore, *ξ* exhibits a characteristic double peak shape (Fig. 1c). This is understood recalling the Gaussian intensity profile *I*(*z*): At central point the fluctuating membrane is detected only via small intensity variations, while at the inflection point (IP), the membrane motion is reflected by a large range of intensities. As a consequence, the sensitivity of membrane fluctuation detection is highest at IP.

### DODS geometry

To relate measured intensity flickers to membrane fluctuations quantitatively, the first step is to determine the axial intensity profile *I*(*z*) of the confocal volume. Formally this is given by the Gaussian function


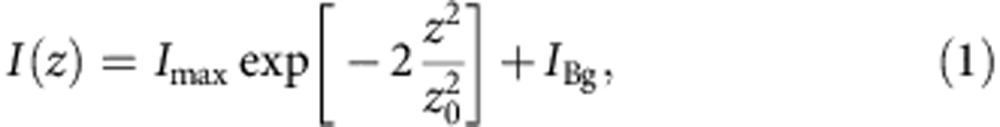


where 
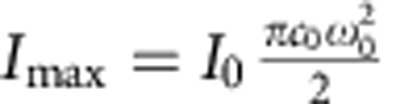
 is the maximal intensity detected at the centre, *z* is the average membrane position within the confocal volume, *I*_Bg_ is the background intensity, *c*_0_ is the fluorophore concentration and *ω*_0_ and *z*_0_ are the radial and axial radii of the confocal volume, respectively. *ω*_0_=280±5 nm and *z*_0_=1,285±10 nm are measured independently using fluorescent beads of sub-resolution size (*N*=10, data taken from two different experiments; all errors given are s.d., throughout, *N* is the number of measured objects which can be beads, GUVs or cells; see Supplementary Fig. 1 for an example of the shape of the confocal volume). Scans in the vertical direction confirm the Gaussian profile of *I*(*z*) in all experimental cases, which is then fitted using equation (1) (Fig. 1a–c).

**Figure 2:**
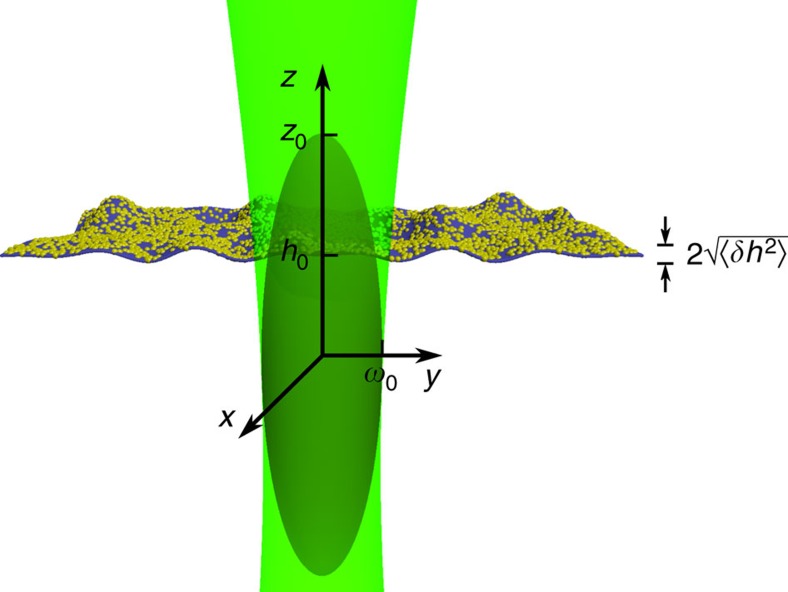
Sketch of the membrane-confocal volume set-up for DODS. The coordinate system and notations are defined. The membrane is in the *x*–*y* plane and undergoes small displacements in the *z* direction. The membrane is positioned such that its average *z* position, *h*_0_, is at the inflection point of the intensity distribution, *I*(*z*). *ω*_0_ and *z*_0_ are the radii of the beam waist in the radial and axial directions, respectively.

For a fluctuating membrane, the instantaneous vertical position *z* is time dependent and defined as *z*=*h*(*t*)=*h*_0_+*δh*(*t*). *h*_0_≡〈*h*(*t*)〉 is the mean membrane position and *δh*(*t*) is the instantaneous membrane fluctuation (see Fig. 2). The relation between intensity and membrane fluctuations, *δI*(*t*) and *δh*(*t*), respectively, is obtained by expanding *I*(*z*) in equation (1) around *h*_0_ up to second order. At IP, which is the optimal position for membrane fluctuation measurements (Fig. 1c,g,k), the second derivative of *I*(*h*_0_) vanishes and the relation simplifies to:


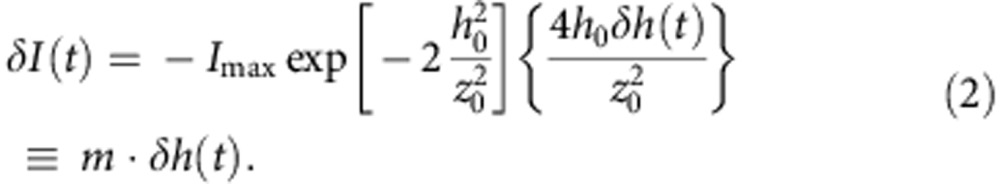


Equation (2) can be used to calculate *δh*(*t*) from *δI*(*t*) via the slope *m* at IP, while *m* is determined in each measurement from the Gaussian intensity profile *I*(*z*). Subsequently, the displacement autocorrelation function (dACF), 〈*δh*(*τ*)*δh*(0)〉, is either built from the calculated *δh*(*t*), or, equivalently, calculated directly from the recorded ACF through the relation:


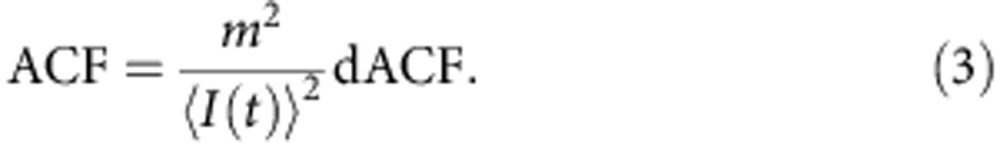


Detailed derivation is given in Supplementary Note 1. Note that use of the experimentally determined slope *m*, rather than a theoretical factor derived from system parameters, ensures that aberrations and image imperfections due to optical inhomogeneity, which may be present in complex systems like nucleated cells, are automatically accounted for.

### Axial and temporal resolution

The axial resolution, which is the smallest displacement that we can measure, is related to the shape of the confocal volume and the background noise and was determined to 20 nm by the following three approaches: (1) the spatial error introduced via the linear intensity-height approximation was checked using Taylor expansions of second or third order in *δh*(*t*) for the conversion of intensity to membrane fluctuations. By comparison of the resulting traces with the linear expansion in *δh*(*t*), a maximal error of 12 nm for the average stochastic displacement was found (see Supplementary Note 2 for examples and further details). (2) The axial resolution in the *z* direction can be calculated from data representing the detection limit, for example, bending fluctuation traces from tensed GUV or supported lipid bilayer (SLB) (see Supplementary Figs 2 and 3). Here, the maximum value of the correlation amplitude amounted to *ξ*_det_=0.0024. At the IP, for a typical slope of *m*=130 k.c.p.s. μm^−1^ (k.c.p.s., kilo counts per second) and intensities of 50 k.c.p.s., equations (2) and (3) yield an axial resolution limit of 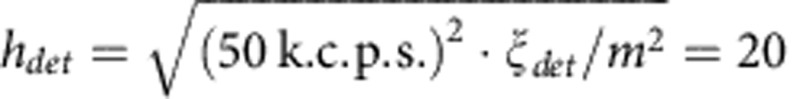 nm. (3). The influence of the signal-to-noise ratio on the axial resolution is estimated theoretically, calculating the error in fluctuations Δ*h* for variable signal and background intensities. Here the conversion of intensities to *δh*(*t*) at the IP is assumed. An increase in background intensity by Δ*I*_Bg_ and with the slope 
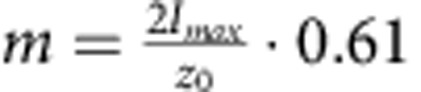
, then leads to the spatial error:


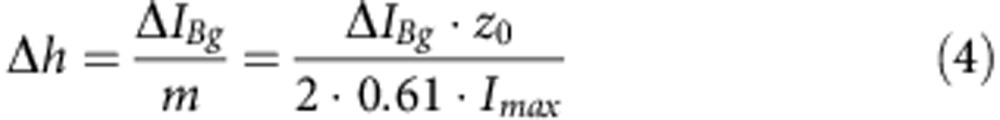


Supplementary Fig. 4 shows the result of this calculation for fluorophore intensity counts between *I*_max_=0–90 kcps and background intensities varying between *I*_*Bg*_=0–9 kcps. A lower limit of Δ*I*_*Bg*_ is given by the dark current of the avalanche photodiode detection amounting to 0.5 kcps. This is also a common background intensity for the experiments presented here. Clearly, for signal intensities of 50–90 kcps the error is well below 20 nm.

It should be noted, that other systems may exhibit higher Δ*I*_*Bg*_ values, resulting in a corresponding drastic increase in the spatial error and any uncorrelated background signal will further dampen the amplitude of the autocorrelation curve. For example, utilization of phenol red containing medium raised Δ*I*_*Bg*_ to 2 k.c.p.s. and increased the error in *h* to >20 nm. Its use was therefore avoided and care was taken to work with minimal possible background intensities.

The temporal resolution of DODS was determined to 10 μs, despite the fact that raw data are recorded at the rate of 200 ns per point. However, to obtain a reasonable signal-to-noise ratio, with uncertainties in the dACF shape well below the axial resolution, we integrate over 10 μs and record for a minimum of 25 s (see Supplementary Fig. 5 for a detailed analysis).

### Validation and application to GUVs

**Figure 3:**
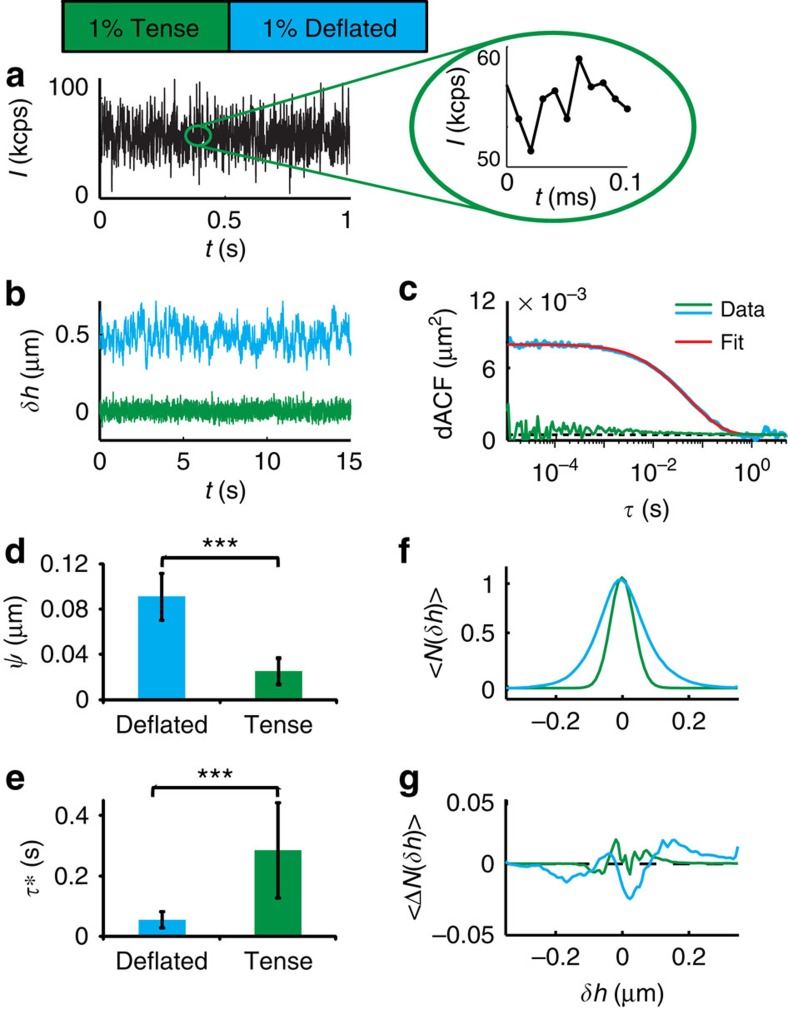
DODS validation and application to GUVs. (**a**) A typical intensity trace recorded at IP (inset: zoom). (**b**) Displacement trace, *δh*, for tense and deflated GUV (the latter is shifted by +0.5 μm for clarity) and (**c**) corresponding dACF. Fitting of dACF (red line) yields the fluctuation amplitude, 
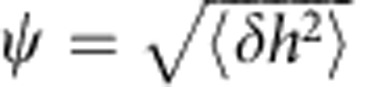
, and the relaxation time, τ*, which define the membrane tension and the viscosity of the surrounding medium (see text for details). (**d**) 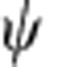 and (**e**) τ* for deflated and tense GUVs. Significance levels, *P*, were evaluated by Mann–Whitney *U*-test: *P*<0.001 (***), *P*<0.01 (**) and *P*<0.05 (*). Error bars denote s.d. of results. (**f**) Histogram of membrane fluctuations, 〈*N*(*δh*)〉, and (**g**) deviation from a Gaussian fit, 〈Δ*N*(*δh*)〉. Data represent the average of all tense (*N*=10, 3 experiments) and deflated (*N*=46, 9 different experiments) vesicles, respectively.

We validated DODS by comparing bending fluctuations of model membranes with well-known theoretical predictions^28^,^29^, previous experimental results^20^,^22^,^28^,^29^ and comparison to dual-wavelength RICM (DW)–RICM^17^,^44^; see Supplementary Note 3). The amplitude of fluctuation 
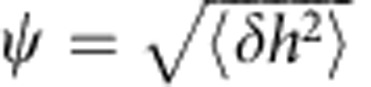
 and the relaxation time τ* were obtained from dACFs measured at the distal surface of GUVs, which were also fitted with setup-specific theoretical expressions to obtain the membrane tension and viscosity (see Supplementary Note 4, Supplementary Equation (9))^28^. As expected, the fluctuations were stronger for the deflated GUVs (90±20 nm, *N*=46), as compared with the tense GUVs (25±4 nm, *N*=10) with an opposite trend for τ* (0.06 s for deflated and 0.28 s for tense, see Fig. 3). Significance levels were determined by Mann–Whitney *U*-test, reported as *P*<0.001 (***), *P*<0.01 (**) and *P*<0.05 (*).

To test whether the method reflects the bending fluctuations of GUVs correctly, we extracted the trace δ*h* over an interval of 60 s and plotted the probability distribution function of bending fluctuations, *N*(*δh*). For all GUV of a specific condition, this was then averaged, yielding 〈*N*(*δh*)〉. In both, deflated and tense case, the fluctuation distribution was nearly Gaussian, as is expected for the case for a linear system under equilibrium. Deviations from the Gaussian were quantified by skewness (*S*), which is a measure of the asymmetry of the probability distribution, and kurtosis (*K*), which yields a measure of the ‘peakedness' of the probability distribution. Here only slight deviations from zero (*S*=0.5±0.3, *K*=−0.8±0.2 for deflated GUVs and *S*=0.2±0.2 and *K*=−0.7±0.2 for tense GUVs; see Fig. 3) were obtained. Fitting of the dACF with Supplementary Equation (9) of Supplementary Note 4, yields the average tension and viscosity. The tension amounts to *σ*=0.5±0.3 μJ m^−2^ for deflated GUV, which agrees well with literature^19^,^20^,^29^. The viscosity (1.2±0.6 mPas) is, as expected, close to that of water (1.0 mPas) with a slight deviation arising from the higher viscosity of the sucrose solution inside the vesicle *η*=1.4 mPas. Comparative measurements of DODS and DW-RICM of the vesicle membrane near the substrate yield fluctuation values in high agreement for both techniques (see Supplementary Note 3). Amplitudes for fluctuations near the substrate amount to 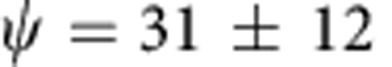 (DODS) and 26±21 nm (DW-RICM). In summary, bending fluctuations measured with DODS are fitted very well with existing theory and yield reliable values for fluctuation amplitudes, tension and dissipation. Having established DODS as an accurate and reliable technique, (see Supplementary Fig. 2 and Supplementary Note 2 and 5 for further specification and control measurements), we then applied it to progressively more complex systems—first RBCs and then nucleated cells.

### Application to RBCs

**Figure 4:**
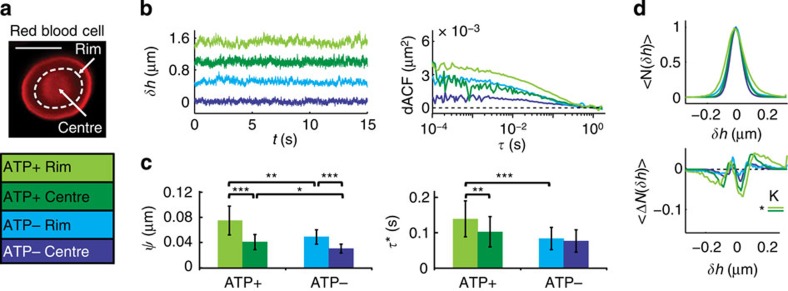
DODS applied to RBCs. (**a**) Fluorescence image and measurement positions (white dashed lines) for a RBC, scale bar, 5 μm. (**b**) Displacement traces, *δh*, (shifted by +0.5 μm for clarity) and dACFs (rim/centre, ATP+/ATP−). (**c**) Fluctuation amplitude, 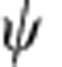, and relaxation time, τ*. Error bars denote s.d. (**d**) Histogram of displacements, 〈*N*(*δh*)〉, and deviation from a Gaussian fit, 〈Δ*N*(*δh*)〉, averaged for all cells (seven different ATP+ experiments (rim: *N*=31, centre: *N*=24) and seven different ATP− experiments (rim: *N*=26, centre: *N*=18)). Significance levels for differences in skewness (*S*) and kurtosis (*K*) are indicated. Significance according to Mann–Whitney *U*-test *P*<0.001 (***), *P*<0.01 (**) and *P*<0.05 (*).

RBCs are simple cells known to undergo bending fluctuations with a still debated contribution from active processes^24^,^25^. They exhibit a biconcave shape maintained by ATP powered remodelling of their membrane coupled spectrin network. In recent years, ATP has been proposed to also facilitate non-equilibrium dynamic fluctuations of the RBC membrane.^24^,^25^,^26^ To further elucidate this aspect, we performed DODS measurements at the centre and at the rim of human RBCs, in the presence or absence of ATP (Fig. 4a,b). Fluctuation amplitudes are significantly larger at the rim (50±10 nm, *N*=26) in comparison to the centre (30±10 nm, *N*=18) and increase by a factor of 1.4 in the presence of ATP (rim: 74±10 nm, *N*=31, centre: 41±10 nm, *N*=24) in agreement with Tuvia *et al*.^45^ and Park *et al*.^25^ (Fig. 4c). The fluctuations relaxed much faster in ATP depleted cells, where only thermal fluctuations are expected (τ*=0.09±0.01 s (centre) and 0.10±0.01 s (rim)). Extracting the probability distribution function of the membrane fluctuations, ATP−cells, exhibited a Gaussian distribution, as expected. Skewness and kurtosis values (Fig. 4d, *S*=0.1±0.5 (rim)/0.3±0.6 (centre) and *K*=−1.2±0.3 (rim)/−1.0±0.9 (centre)) were comparable to vesicles. For ATP+ cells, the same was found at the centre, but unexpectedly, a deviation from a Gaussian distribution was found at the rim. As characterized by a non-zero skewness and kurtosis, this indicates a nonlinear behaviour of the system (*S*=0.1±0.6 (rim)/1.1±0.6 (centre) and *K*=−1.9±0.6 (rim)/−0.9±0.3 (centre)).

Taken together, our results suggest that the RBC membrane is a metabolically regulated active structure, whose motion is dominated by thermal contributions, with residual ATP driven activity detectable at the rim of the cell.

### Application to macrophages

**Figure 5:**
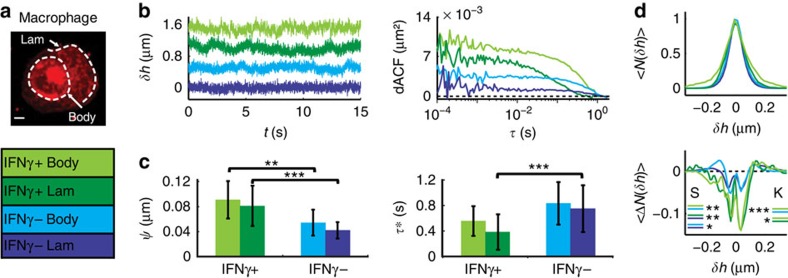
DODS applied to macrophages. (**a**) Fluorescence image and measurement positions (white dashed lines) for a macrophage, scale bar, 5 μm. (**b**) Displacement traces, *δh*, (shifted by +0.5 μm for clarity) and dACFs (cell body (body)/lamellipodium (lam), IFNγ+/IFNγ−). (**c**) fluctuation amplitude, 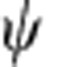, and relaxation time, τ*. Error bars denote s.d. (**d**) Histogram of displacements, 〈*N*(*δh*)〉, and deviation from a Gaussian fit, 〈Δ*N*(*δh*)〉, averaged for all cells (six different IFNγ+ experiments (lam: *N*=29 and body: *N*=11) and five different IFNγ− experiments (rim: *N*=26 and centre: *N*=26)). Significance levels for differences in skewness (*S*) and kurtosis (*K*) are indicated. Significance according to Mann–Whitney *U*-test *P*<0.001 (***), *P*<0.01 (**) and *P*<0.05 (*).

Next we applied DODS to a complex cell and measured a change in stochastic displacements as a response to a biologically relevant stimulus: qualitative measurements on cells have been undertaken before, yet, so far it was not possible to quantify stochastic bending fluctuations to this accuracy. As an example, we probed the priming of human macrophages in response to stimulation by the soluble cytokine interferon gamma (IFNγ). IFNγ is known to augment many host-defense functions in macrophages and through its interplay with membrane bound activators it increases actin polymerization and ruffling activity^46^. With DODS, membrane dynamics were quantified measuring the stochastic displacements at the lamellipodium (lam) and at the edge of the cell body (body) (Fig. 5a). Correlation analysis was carried out for non-stimulated (IFNγ−, *N*=26 lam, *N*=26 body) and stimulated cells (IFNγ+, *N*=29 lam, *N*=11 body). IFNγ− macrophages exhibit comparable fluctuation amplitudes of about 45 nm at the lam and the body (40±10 nm (lam) and 50±20 nm (body)) (Fig. 5b). As expected, IFNγ treatment enhanced the fluctuation amplitude (80±30 nm (lam) and 90±30 nm (body), respectively, for IFNγ+ cells). τ* decreased from 0.8±0.4 s (both lam and body) in untreated cells to about 0.5 s in primed cells (0.4±0.3 s and 0.6±0.2 s), which is opposite to the trend of enhanced fluctuations in RBCs (Fig. 5c). The stochastic membrane displacements in IFNγ− cells are nearly Gaussian (*S*=−0.9±0.8 (body)/0.8±0.5 (lam) and *K*=−1.0±0.3 (body)/−0.8±0.4 (lam)), but stimulation significantly increases both skewness and kurtosis indicating vastly increased active contributions (Fig. 5d, *S*=3.4±1.0 (body)/3.8±1.0 (lam) and *K*=−5.1±1.1 (body)/−2.4±0.7 (lam)). Hence, our data suggests that amplitude, time correlation and the fluctuation distribution function are indicative of the cell's biological state.

## Discussion

In this study, a new technique to quantify membrane fluctuations with unprecedented spatiotemporal accuracy was set-up. This technique, DODS, is a novel tool for basic research and future biomedical applications, and is particularly valuable as it measures stochastic displacements in living cells and, unlike existing techniques^21^,^25^, does so at any chosen part of the cell. Crucially, the relation between displacement and intensity is experimentally determined, thus no assumptions regarding the optics of the cell have to be made.

DODS was applied to the membranes of GUVs, RBCs and human macrophages. Tension (0.5 μJ m^−2^) and viscosity (1.2 mPas) relevant to a freely fluctuating model membrane were determined for the first time. Previous attempts to measure bending fluctuations with enhanced accuracy were either limited by camera detection and substrate proximity^20^ or led to an unreasonable high viscosity exceeding the expected value by × 20 (ref. 24). The stochastic displacements of RBC membrane is a highly debated topic and our data contribute to the closure of the ongoing discussion on RBC membrane activity. Our proof-of-principle demonstration of the stochastic displacements of the plasma membrane of macrophages gives access to an entirely new kind of data that should provide insight into hitherto inaccessible stochastic cellular processes as well as may stimulate further theoretical development.

The axial resolution of DODS in the *z* direction, which is currently estimated to be 20 nm, can be improved using lateral instead of axial scans. In the lateral direction (*x*–*y* plane), the intensity Gaussian is narrower and simultaneously the slope at the IP is steeper. More precisely, at the IP, where *z*_0_=±2*h*_0_ and equation (2) yield 
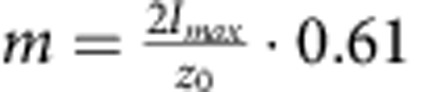
, lateral scans suggest a resolution enhancement by a factor 4, due to the exchange of *z*_0_ for *ω*_0_∼*z*_0_/4. Similarly, it may be improved by an order of magnitude using stimulated emission depletion (STED)–FCS^34^,^38^, where the effective beam size is much smaller and therefore the intensity Gaussian much narrower. DODS can be extended to larger membrane movements by retaining higher order terms in the intensity-displacement relationship. An interesting extension concerns simultaneous measurement of diffusion and membrane fluctuations by using two different fluorophores at high (for DODS) and low (for FCS) molecular concentration. This has been attempted before by tracing a fluorescent molecule at low concentration close to a membrane exhibiting bending fluctuations^47^. However, a direct evidence separating the two contributions is still missing. Diffusion and membrane fluctuations are speculated to be correlated for model membranes^48^ and may provide crucial data in the cellular context by linking membrane tension to molecular dynamics^49^. With axial resolution comparable to the established super-resolution techniques, and time resolution comparable to FCS, DODS will take advantage of ongoing improvements to FCS^34^,^38^,^39^,^40^,^41^ and can be expected to be established as a companion technique to FCS-based diffusion and structural organization measurements^31^, thus linking membrane scale phenomena to molecular scale events.

## Methods

### Data acquisition and analysis

DODS measurements were undertaken at a confocal laser scanning microscope (LSM710, Zeiss) equipped with a 5-mW HeNe laser (*λ*=543 nm) and two avalanche photodiodes (Perkin Elmer, Waltham, MA, USA) for photon counting detection. Appropriate filter sets were chosen (for the dyes used here: beam splitter 488/543 nm for excitation and 580-nm long pass filter for emission, Zeiss) and a water immersion objective (C-Apochromat, × 40, numerical aperature 1.2, Zeiss) was used. Data acquisition and realtime autocorrelation were executed via the software ZEN (version 2008, Zeiss). Before the start of a DODS measurement, fluorescence and phase contrast images were acquired to determine the overall shape of the GUV or the cell and to choose the appropriate position. In a typical experiment, with the fluorescent membrane positioned inside the confocal volume, the excitation laser is attenuated until a maximum count *I*_max_ of ∼70–100 kcps is reached. Subsequently, a fast scan perpendicular to the membrane is recorded—this establishes the parameters of the Gaussian intensity distribution of the illuminating volume. The confocal spot is then positioned such that the mean position of the membrane is at the IP of the Gaussian (given by the intensity *I*_IP_=0.61·*I*_max_). Intensity fluctuations are recorded for a specific time interval and the ACF is calculated. Tests were undertaken to ensure that at the applied excitation intensities bleaching was negligible and that fluorophore response was linear. Ergodicity and stationarity of data were tested by calculating the deviation between the ACF over the total recording time and the ACF over a subset of 5 × *x* s, x∈{1–10} duration (Supplementary Note 2). Twenty-five seconds were determined to be minimal recording time *t*_min_, which ensures ergodicity and the complete build-up of the dACF. For GUVs and RBCs data were recorded for 2–3 min. In case of macrophages, cell movement limited measurement times to 40–120 s, which is nevertheless >>*t*_min_ and therefore sufficient to build the ACF.

To compare DODS with a different technique, identically prepared GUV were measured with DODS and DW-RICM^17^,^44^. This microinterferometric technique can measure the height of a fluctuating membrane above a substrate with 4-nm axial resolution and 50-ms temporal resolution (as limited by the camera speed). The RICM image is formed from interfering rays being reflected at different optical layers in the sample. Depending on the difference of their optical path length, these rays interfere in a destructive or constructive manner. Knowing the refractive indices and thicknesses of the optical interfaces, the membrane-substrate height is calculated. For DW-RICM, two images (for *λ*=546 nm and 436 nm) are simultaneously recorded. This allows to unambiguously determine membrane-substrate heights up to several ∼100 nm. Images were acquired with a DW-RICM set-up described in detailed before^17^,^22^,^44^: an inverted microscope (Zeiss Axiovert 200, Carl Zeiss, Göttingen, Germany) equipped with a filter cube with crossed polarizers and a × 63 Antiflex Plan-Neofluar oil objective with a numerical aperture of 1.25 and built in lambda quarter plate was used. Light emitted by a metal halogenide lamp (XCite, Exfo, Quebec, Canada) was filtered using a dual-interference filter (*λ*=546±10 and 436±20 nm). The numerical aperture of illumination was set to 0.54. To achieve maximum contrast the antiflex technique was applied. To record two micrographs simultaneously, the reflected light was split according to its wavelength (FT 460 nm, LP 470 nm (Carl Zeiss, Göttingen, Germany) and BP 436±10 nm (AHF, Tübingen, Germany)) and focused on two separate digital CCD cameras (sensicam qe, PCO, Kehlheim, Germany). Image recording was controlled by the software OpenBox (version 1.77, Informationssysteme Schilling, Munich, Germany).

Images and data were analysed using the self-written routines in Matlab (version 3.0 (R2010b), The MathWorks, Inc. MA, USA) using the image processing toolbox and ImageJ (version 1.45 s, Rasband, W.S., NIH, Bethesda, MD, USA). Errors reported throughout are s.d.

### Substrates

Thickness corrected glass coverslips (d=170±10 μm, Assistent, Karl Hecht KG, Sondheim, Germany) were cleaned by the following detergent treatment: ultrasonication in 2% Hellmanex solution (Hellma, Müllheim, Germany) for 10 min, flushing thoroughly with ultrapure water produced by a water purification system (Milli-Q Gradient A10, Millipore, San Francisco, CA) and again ultrasonication (2 × 10 min) in ultrapure water followed by repeated flushing with ultrapure water. To prevent unspecific interactions in GUV and RBC experiments, bare glass was passivated by incubation with 5 mg ml^−1^ bovine serum albumin (BSA, Sigma, Saint Louis, MO, USA) for 15 min. For macrophages, cell culture dishes (3-cm diameter, Greiner, Solingen, Germany) with thickness corrected glass coverslips were pre-coated with 10 μg ml^−1^ human fibronectin (BD Biosciences, Bedford, USA) in PBS for 30 min at 37 °C. In all cases, excess protein was removed by exchanging the buffer in a series of 10 washing steps.

### Fluorophores

Fluorophores were purchased from Invitrogen (Eugene, OR, USA) as lipid conjugates. For GUVs, tetramethylrhodaminethiocarbamoyl conjugated to the lipid DHPE (*N*-(6-tetramethylrhodaminethiocarbamoyl)-1,2-dihexadecanoyl-sn-glycero-3-phosphoethanolamine, triethylammonium salt) was used at 1 mol% for DODS measurements. For cells, the fluorophore of choice was Texas Red, again conjugated to DHPE (Texas Red-1,2-dihexadecanoyl-sn-glycero-3-phosphoethanolamine, triethylammonium salt). The cell membrane was labelled as described below.

### Lipids

SOPC (1-stearoyl-2-oleoyl-sn-glycero-3-phosphocholine), DOPE-PEG2000 (1,2-dioleoyl-sn-glycero-3-phosphoethanolamine-*N*-(methoxy(polyethyleneglycol)-2000)), DOPE-cap-biotin (1,2-dioleoyl-sn-glycero-3-phosphoethanolamine-*N*-(cap biotinyl)), DOPE (1,2-dioleoyl-sn-glycero-3-phosphoethanolamine) and DOTAP (1,2-dioleoyl-3-trime-thylammonium-propane, chloride salt) were purchased from Avanti Polar Lipids (Alabaster, AL, USA) and were used as it is.

### Giant unilamellar vesicles

GUVs made of SOPC with 2 mol% DOPE-PEG2000, 5 mol% DOPE-cap-biotin and 0.001–1 mol% DHPE-tetramethylrhodaminethiocarbamoyl were electro-swollen in 230 mOsm l^−1^ sucrose solution as reported in ref. 22. Therefore, 20 ml of a solution of the lipid dissolved in chloroform (2 mg ml^−1^) was dispersed on glass slides coated with indium tin oxide (PGO, Iserlohn, Germany) and the solvent desiccated under vacuum overnight. Two lipid-coated glass slides were mounted in a teflon chamber filled with 230 mOsm l^−1^ sucrose solution, at a distance of 1 mm. An alternating voltage of 1.7 V and 10 Hz was applied for 1.5 h. This yielded GUVs of about 40-μm average diameter. The concentration of the fluorescent lipids depended on the experiment, 0.001% being appropriate for FCS and 1% for DODS. Observations were done in PBS buffer (10 mM Na_2_HPO_4_, 2 mM KH_2_PO_4_ and 3 mM KCl) at pH 7.2, with added NaCl to obtain the desired osmolarity (all salts were from Sigma, Germany). PBS (400 mOsm l^−1^) was used for experiments with floppy GUVs and 250 mOsm l^−1^ for tense GUVs. In a typical experiment, vesicle solution in 1:50 dilution was added to the observation buffer in the experimental chamber, which was then covered with a glass slide to avoid osmolarity changes due to evaporation. DODS measurements were started 30 min later, to ensure full equilibration of the system. All measurements were undertaken at room temperature.

### Preparation of RBCs

Human RBCs were freshly prepared before each experiment by pricking the finger of a healthy donor and then diluting 10 μl blood in 1 ml PBS (pH 7.4, 300 mOsm·l^−1^, see above). RBCs were washed twice with PBS by centrifugation (1 min, 200*g*, Eppendorf Centrifuge 5804R, Hamburg, Germany) and aspiration of the supernatant. After fluorescent staining of the cell membrane (see below), cells were centrifuged again and the pellet was diluted 1:17 in the observation buffer (PBS+0.1 mg ml^−1^ BSA for ATP depleted and PBS+0.1 mg ml^−1^ BSA+10 mM D-Glucose for ATP saturated cells). In case of ATP depletion, cells were kept in the observation buffer for 24 h before membrane staining and subsequent imaging.

### Preparation of macrophages

Primary human macrophages were grown at 37 °C and 5% CO_2_ in Roswell Park Memorial Institute Medium (RPMI 1640, Gibco, Karlsruhe, Germany), with 10% (v/v) fetal bovine serum (Sigma), 1:100 penicillin/streptomycin (10,000 U ml^−1^, Sigma) and without phenol red, henceforth referred to as RPMI^+^. Before measurements cells were washed with PBS to withdraw surplus organic compounds and medium. Subsequently, cells were incubated in 3-ml trypsin-EDTA solution (consisting of 0.5% trypsin and 0.2% EDTA, Sigma) for 3 min at 37 °C. This yielded cells in suspension, which were centrifuged (200*g*, 3 min), collected and resuspended in the experimental buffer (RPMI^+^ for resting or RPMI^+^+1,000 U·ml^−1^ IFNγ for primed cells). After seeding in the measurement chamber and incubation for ≥24 h the cell plasma membrane was stained as described below and imaging was started subsequently. During measurements a microscope with incubator at 37 °C and CO_2_ supply was used.

### Fluorescent labelling of cells

Fluorescent labelling of RBC or macrophage plasma membranes was carried out by a method developed by Csiszár *et al*.^50^ Therefore, fusogenic liposomes consisting of ternary lipid mixtures, enabled highly efficient incorporation of fluorescent molecules into mammalian cell membranes. Small multilamellar liposomes were produced from a 1 mg ml^−1^ mixture of DOPE, DOTAP and Texas Red—DHPE in a 1:1:0.2 weight ratio. To do this, the lipid were first dissolved in chloroform and the solution was then dried under vacuum for 20 min. The lipids were resuspended in 20 mM 2-(4-(2-hydroxyethyl)-1-piperazinyl)-ethane-sulfonic acid (pH 7.5, HEPES, VWR) at 2.2 mg ml^−1^ final concentration. To produce multilamellar liposomes the suspension was stirred 1–2 min on a vortex-mixer followed by 10-min ultrasound bath treatment. The liposomes were stored at 4 °C. Cells were incubated with the liposome solution in a 1:100 dilution for 12 min at 37 °C. They were then washed and resuspended in fresh experiment buffer or medium, as appropriate.

## Additional information

**How to cite this article:** Monzel, C. *et al*. Measuring fast stochastic displacements of bio-membranes with dynamic optical displacement spectroscopy. *Nat. Commun.* 6:8162 doi: 10.1038/ncomms9162 (2015).

## Supplementary Material

Supplementary Information Supplementary Figures 1-8, Supplementary Notes 1-5 and Supplementary References
