# Supplementary material for: Measuring fast stochastic displacements of bio-membranes with dynamic optical displacement spectroscopy
Source: Nat Commun. 2015 Oct 6;6:8162. doi: 10.1038/ncomms9162 (PMC4600712; doi:10.1038/ncomms9162)
Supplement: Supplementary Information — Supplementary Figures 1-8, Supplementary Notes 1-5 and Supplementary References [file ncomms9162-s1.pdf]

Supplementary Figure 1: Confocal detection volume

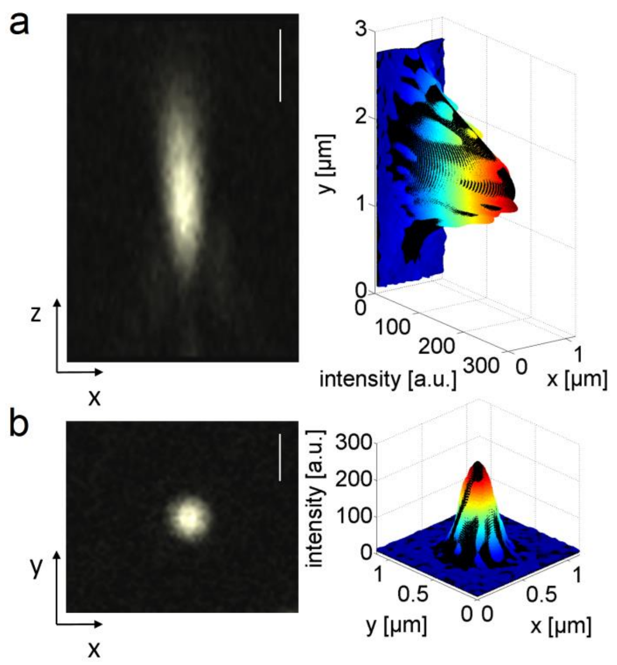

Knowledge about the intensity distribution in the confocal volume and its size is crucial for DODS measurements: To verify the near Gaussian shape in both axial as well as lateral direction, the confocal detection volume was measured for $\lambda = 543$ nm with beads of subresolution size. (a) Left: Axial (xz) view of measured bead intensity. Right: The intensity distribution is fitted with a 2D asymmetric Gaussian (rgb colour). (b) Left: Lateral (xy) view of measured bead intensity. Right: Fit of 2D symmetric Gaussian (rgb colour).
Scale bars: 1 µm (xz) and 0.5 µm (xy).

Supplementary Figure 2: Axial intensity scans and ACFs for GUVs

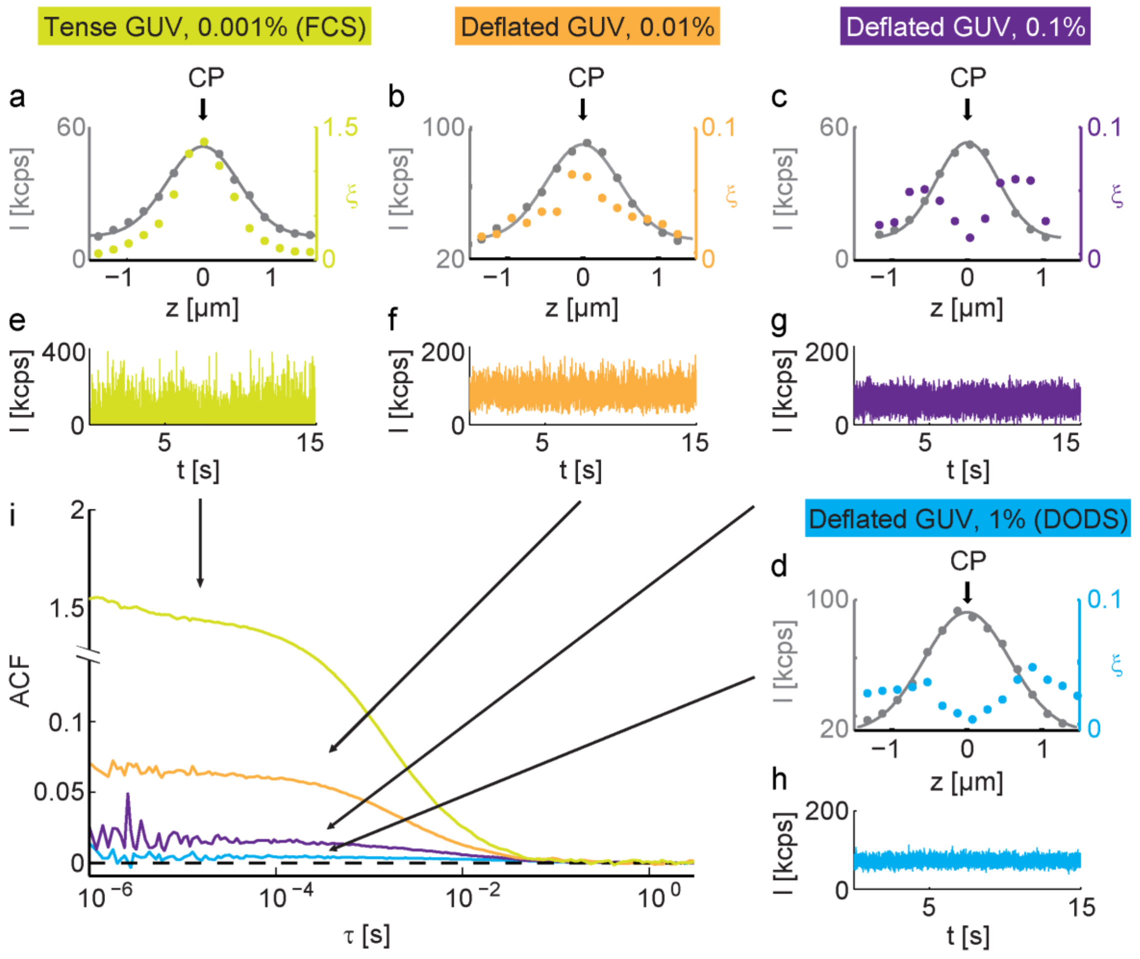

(a)-(d) Recorded axial scans of GUVs with different fluorophore concentrations. Average intensities (grey dots), Gaussian fit (grey line) and correlation amplitude ξ for fluorophore concentration 0.001% (yellow), 0.01% (orange), 0.1% (purple) and 1% (light blue) are shown. ξ changes from a single-peaked to a double-peaked shape when the GUV is deflated and the fluorophore concentration increased. (e)-(h) Intensity traces $I(h(t))$ were measured at the center CP of the confocal volume. (i) The ACF, defined as $\langle \delta I(\tau)\,\delta I(0)\rangle / \langle I(t)\rangle^2$, is calculated from these traces and exhibits a flattening of the amplitude.

Supplementary Figure 3: Axial intensity scans and ACFs for SLB

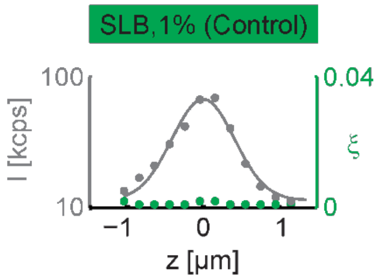

Recorded axial scan of a supported lipid bilayer (SLB) as control measurement. Average intensities (grey dots), Gaussian fit (grey line) and correlation amplitude ξ (green dots). ξ remains flat over the whole scan. Since a SLB cannot have spatial fluctuations, the maximal value, $\xi_{\mathrm{det}} = 0.0024$, marks the detection limit of our DODS setup.

Supplementary Figure 4: Influence of the signal-to-noise ratio on spatial resolution

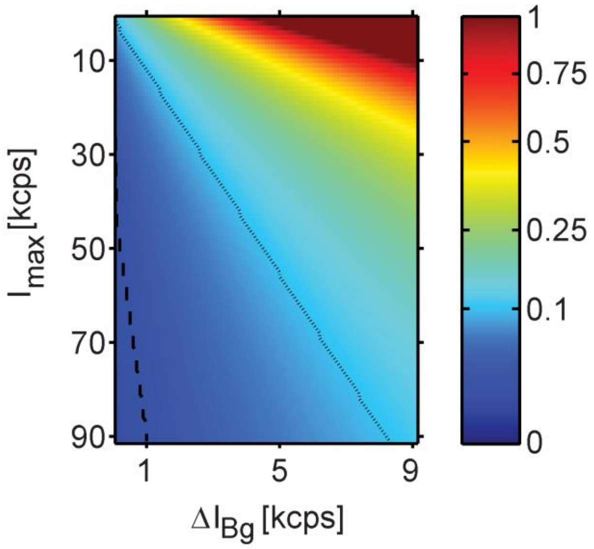

Illustration of typical signal intensity $I_{\max}$ vs. background intensity ΔI_{Bg}. The colour code indicates the corresponding spatial error Δh in µm, which is estimated analytically (see text for details). Black dashed line marks the $\Delta h = 20$ nm and dotted black line the $\Delta h = 100$ nm error.

Supplementary Figure 5: Stationarity of measurement

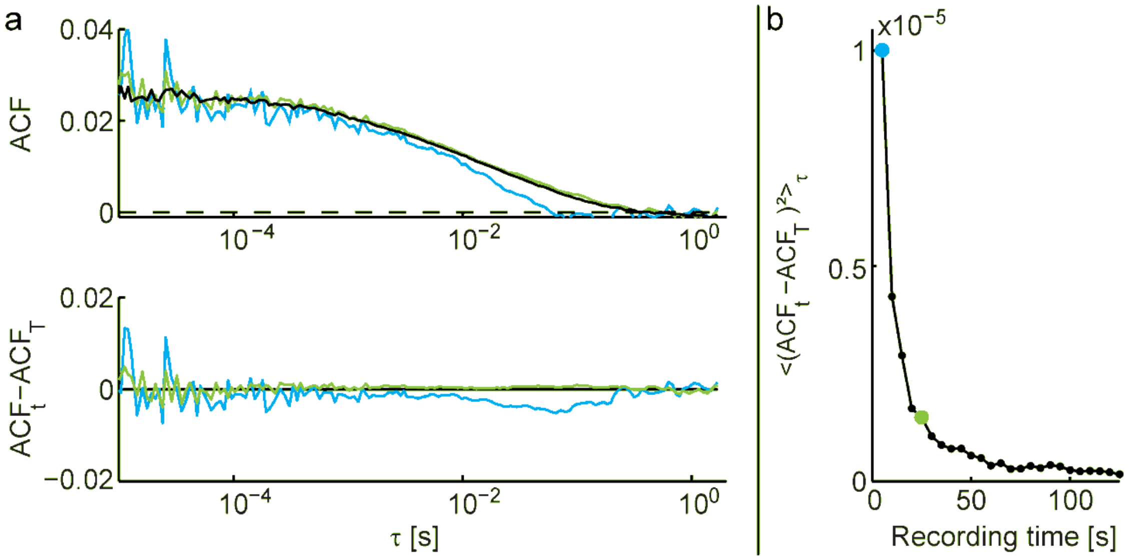

GUV membrane fluctuations are measured at the IP for a total recording time T = 3 min. The signal is subdivided into 5 s intervals, so-called repeats, and an ACF for each is calculated. (a) Top: Average ACF derived from all repeats ($36 \times 5\,\mathrm{s}$ = 3 min, black), in comparison to single repeat (5 s recording time, blue) and five repeat average (25 s recording time, green). Bottom: Deviation between the ACF of total recording time T = 3 min and the ACF for t = 5 s (blue) or t = 25 s (green). (b) Square deviation averaged over all lag times τ vs. recording time (5 s (blue) and 25 s (green) are indicated). With increasing recording time, the shape of the ACF stabilizes.

Supplementary Figure 6: Theoretical correlation amplitude

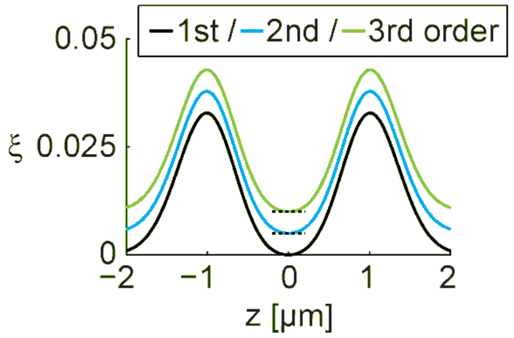

Here, ξ is shown for different orders of the approximation for small fluctuations $\delta h / z_0 << 1$. Parameters used to generate the curves are: $I_{max} = 60\,\mathrm{kcps}$, ω_0 = 0.281 µm and ω_0 = 1.284 µm and the membrane parameters (see also **Supplementary Note 3**) $\kappa = 20$ k_BT, $\sigma = 0.5$ µJm^{-2}. For visibility reasons, curves are shifted along the ξ-axis by 0.005. ξ is the same for all orders of approximation and exhibits the characteristic double-peaked shape, as observed in DODS experiments.

Supplementary Figure 7: Error in height conversion

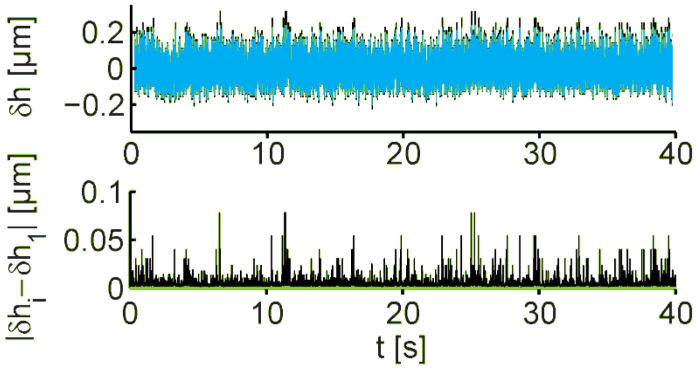

Top: displacement traces of a GUV membrane at the GUV distal site. Measurements were recorded at the inflection point of the confocal detection volume and converted via the linear intensity dependence (blue), for the 2nd (green) and 3rd (black) order expansion (see **Supplementary Eq. 2**). Bottom: absolute deviation between higher and first order conversion, $|\delta h_2 - \delta h_1|$ (green) and $|\delta h_3 - \delta h_1|$ (black). The average deviation $\langle |\delta h_3 - \delta h_1| \rangle_t$ amounted to 12 nm.

Supplementary Figure 8: Comparison of DODS and DW-RICM.

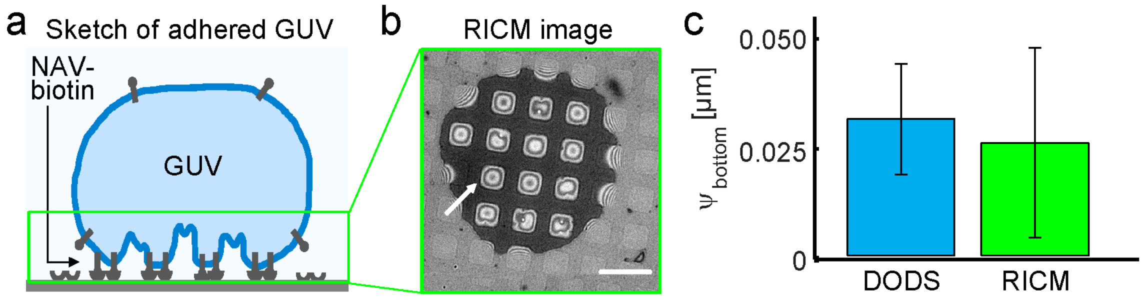

(a) Sketch of GUV adhered to protein grid with adhered and freely fluctuating membrane regions. Note, the sketch is not to scale. (b) One of the two RICM images recorded with DW-RICM showing the membrane bottom region. The arrow points to a square region where fluctuations where evaluated. Scale bar: 10 µm. (c) Average ψ and standard deviation as obtained from measurements at the vesicle bottom.

Supplementary Note 1: Converting intensity flickers to displacements

Here we derive the theoretical expressions linking first the measured intensity flickers $\delta I(t)$ to the real membrane displacements $\delta h(t)$, and second, the intensity autocorrelation function (ACF) to the displacement autocorrelation function (dACF). We assume that the signal from diffusion of fluorophores is negligible, and that the confocal volume has a Gaussian intensity distribution given by

$$I(z) = I_0 \frac{\pi c_0 \omega_0^2}{2} \exp\left[-2\frac{z^2}{z_0^2}\right] + I_{Bg} \quad . \tag{1}$$

Here, z is the average vertical position of the membrane within the confocal volume (see also **Fig. 2** for an explanation of coordinates and symbols), c_0 is the fluorophore concentration, I_{Bg} is the background intensity, and ω_0 and z_0 are the radial and axial dimensions of the confocal volume (ω_0 = 280 ± 5 nm (N = 10) and z_0 = 1285 ± 10 nm (N = 10)[1], as measured using fluorescent beads of sub-resolution size: data not shown). $I_{\max} = I_0 \pi c_0 \omega_0^2 / 2$ is the maximal intensity detected in a vertical scan through the confocal volume. For a fluctuating membrane, the vertical position z becomes time dependent $z \equiv h(t) = h_0 + \delta h(t)$ where $h_0 \equiv \langle h(t) \rangle$ is the mean membrane position and $\delta h(t)$ is the instantaneous membrane displacement (see **Fig. 2**).

Intensity-displacement relation: For membrane displacements that are small compared to the size of the confocal volume ($\delta h(t)/z_0 << 1$) the intensity fluctuation $\delta I(t)$ around the mean value $\langle I(t) \rangle$ is defined as $I(t) \equiv \langle I(t) \rangle + \delta I(t)$, where $I(t)$ is the instantaneous measured intensity. Using **Supplementary Eq. 1** and expanding $\delta I(t)$ around the mean position h_0,

$$\delta I(t) = -I_{\max} \exp\left[-2\frac{h_0^2}{z_0^2}\right] \left\{ \frac{4h_0\, \delta h(t)}{z_0^2} + \frac{2}{z_0^2}\left(1 - \frac{4h_0^2}{z_0^2}\right)\left(\delta h^2(t) - \langle \delta h^2 \rangle\right) + \mathrm{O}(\delta h^3(t)) \right\}. \tag{2}$$

If the membrane is placed at the inflection point of the intensity profile, as is the case with typical DODS measurements, the second order term vanishes and a first order approximation for $\delta I(t)$ is sufficient (see also **Supplementary Note 2** for validation of this approximation). **Supplementary Eq. 2** then reads

$$\delta I(t) = -I_{\max} \exp\left[-2\frac{h_0^2}{z_0^2}\right] \left\{ \frac{4h_0\, \delta h(t)}{z_0^2} \right\} \equiv m \cdot \delta h(t) \, . \tag{3}$$

In a DODS experiment, the slope m is measured from axial scans in the vertical (z) direction as described in the main text. In the form $\delta I(t) = m \cdot \delta h(t)$ **Supplementary Eq. 3** can be used as long as the intensity distribution in the axial scan exhibits a well-defined inflection point.

---

[1] Error-bars are standard deviations. N is the number of measurements.

ACF-dACF relation: If the membrane fluctuates around a constant mean position (i.e. it has no net displacement over time), the system is amenable to correlation analysis. In general, the correlation amplitude and the autocorrelation function (ACF) of the intensity fluctuations are defined as

$$\xi \equiv \frac{\langle \delta I(t)^2 \rangle}{\langle I(t) \rangle^2} \quad \text{and} \quad ACF \equiv \frac{\langle \delta I(\tau)\, \delta I(0) \rangle}{\langle I(t) \rangle^2} \quad . \tag{4}$$

Consequently, in the limit of small membrane fluctuations, the correlation amplitude and the ACF of the intensity fluctuations become (up to second order in $\delta h(t)$)

$$\xi = \frac{I_{\max}}{\langle I(t) \rangle^2} \exp\left[-4 \frac{h_0^2}{z_0^2} \right] \frac{16 h_0^2}{z_0^4} \psi^2 \equiv \frac{m^2}{\langle I(t) \rangle^2} \psi^2 \ , \tag{5}$$

$$ACF = \frac{\xi}{\psi^2} \langle \delta h(\tau)\, \delta h(0) \rangle \equiv \frac{m^2}{\langle I(t) \rangle^2} dACF \ , \tag{6}$$

defining the amplitude of fluctuations $\psi \equiv \sqrt{\langle \delta h^2 \rangle}$ and the displacement autocorrelation function $dACF \equiv \langle \delta h(t)\, \delta h(0) \rangle$. Note again that for DODS the membrane is placed at the inflection point of the Gaussian intensity profile. Here, the intensity profile is the steepest (the slope m is maximum) and thus, the correlation amplitude is the highest. Note, that the above conversion of intensity into heights has only a unique solution as long as fluctuations remain on one side of the Gaussian illumination profile. In all the cases considered here, this condition is easily fulfilled since the fluctuation amplitudes are much smaller than the axial confocal radius $z_0 \cong 1$ µm. The amplitude of the correlation function and hence the amplitude of fluctuations, as well as the relaxation time τ^* defined as the time-point where $dACF(\tau^*) \equiv dACF(0)/e$, is determined from the plot of the $dACF$.

Supplementary Note 2: Spatio-temporal resolution, recording time and validity of linear intensity/displacement relation

Temporal resolution: The raw data is recorded at rate of 200 ns. This is then averaged in the acquisition software (see Methods section) to 1 µs to build the intensity ACF. The absolute time resolution of the system is therefore 1 µs. However, for the systems studied here we found that the signal to noise ratio at such short times were unacceptably low. To determine the appropriate time cut-off, we examined dACF curves from optimal GUV data and chose the time point at which the value of the noise seen in the dACF data (defined as the standard deviation) exceeds 10% of dACF value. This corresponds to a lag time of 10 µs. Thus, 10 µs is the appropriate time resolution in the present set-up.

Spatial resolution: The spatial resolution is related to the shape of the confocal volume and the background noise. A rough estimate can be obtained by considering the traces from SLB presented in **Supplementary Fig. 2**. The maximum value of the correlation amplitude $\xi_{\det} = 0.0024$, marks the detection limit. At the inflection point, for a typical slope of m =

130 kcps/µm, intensity of about 50 kcps, using **Supplementary Eq. 5**, a spatial resolution limit of $h_{\det} = \sqrt{(50\,kcps)^2 \cdot \xi_{\det} / m^2} = 20\,\text{nm}$ is obtained. The influence of the signal-to-noise ratio on the spatial resolution can be estimated theoretically by calculating the error in displacement considering variable signal and background intensity. Here, a conversion of intensities into displacements at the inflection point of the Gaussian intensity profile (see **Supplementary Eq. 1**) is assumed, where $\delta h \propto \delta I$ holds. More specifically, as the background intensity increases by ΔI_{Bg} the error in the displacement Δh is given by the slope $m = 0.61 \cdot 2I_{\max} / z_0$, such that:

$$\Delta h = \frac{\Delta I_{Bg}}{m} = \frac{\Delta I_{Bg}\, z_0}{0.61 \cdot 2I_{\max}}$$

Supplementary Fig. 4 shows the result of this calculation for typical fluorescence intensity values $I_{\max} = 0 - 90\,\text{kcps}$. Background intensities were varied between $I_{Bg} = 0 - 9$ kcps and z_0 = 1.285 µm is the measured axial radius of the confocal volume. A lower limit of ΔI_{Bg} is given by the dark current of the APDs amounting to 0.5 kcps. This is a common background intensity value for vesicle experiments in PBS buffer. It has to be noted, that other systems may exhibit higher ΔI_{Bg} values, resulting in a corresponding drastic increase in the spatial error. Clearly, only signal intensities of $70 - 100$ kcps and the usually measured background intensity of ≤ 1 kcps, allow for an accuracy of ≤ 20 nm. Note that utilization of phenol red containing medium raised ΔI_{Bg} to 2 kcps, its use was therefore avoided.

Stationarity and recording time: Stationarity of the signal and sufficient recording time are essential prerequisites for correlation analysis. To test their validity in DODS measurements, deflated GUVs of high fluorophor concentration were used and intensity traces of ≥ 180 s duration at the IP acquired. Each measurement was subdivided into ≥ 36 short intervals of 5 s duration, so-called repeats. The ACF of each repeat was calculated and, subsequently, average ACFs for an increasing number of repeats were generated. **Supplementary Fig. 5** illustrates examples of ACF, for a single repeat, an average of 5 repeats, as well as the total average ACF of all repeats after 3 min recording time. Stationarity of the signal is tested for by plotting the deviation between the total average ACF and an ACF of shorter recording time. While a symmetric variation around 0 indicates stationarity, the square of this deviation averaged over all τ specifies the shape stability of the ACF. In our setup, stationarity and a shape precision comparable to the experimental error is achieved in case of 5 repeats. Thus, the minimal recording time of DODS measurements is $5 \times 5 = 25\,\text{s}$, which roughly amounts to 1000 correlation times (cf. **Supplementary Fig. 5a**).

Error introduced by linear approximation: The theoretical correlation amplitude, which is defined as the extrapolated ACF at zero lag time $\tau = 0$, is calculated to first order in $\langle \delta h(t)^2 \rangle$, see **Supplementary Eq. 5**. The theoretical correlation amplitude calculated up to different orders of approximation is shown in **Supplementary Fig. 6**. **Supplementary Fig. 7** depicts an example of intensity-displacement conversion via a Taylor expansion up to 1st, 2nd or 3rd order in $\delta h(t)$. The absolute deviation between higher and first order trace is calculated and averaged. Despite the obvious deviation in $|\delta h_3 - \delta h_1|$ the temporal average yields an error of 12 nm in lateral resolution.

Supplementary Note 3: Comparison of DODS and DW-RICM at the vesicle bottom

DODS was validated by comparing thermal fluctuations measured at the vesicle bottom with measurements obtained by Dual-wavelength Reflection Interference Contrast Microscopy (DW-RICM) [SI4, SI5]. This microinterferometric technique is an advanced setup of RICM where two interferograms for different wavelengths are recorded simultaneously. RICM is highly sensitive to membrane-substrate distances and allows reconstruction of membrane topographic shapes with 5 nm axial resolution and some ~ 10 ms temporal resolution (for further details see **Methods** and [SI4, SI5]). Here, this well established technique is used to compare DW-RICM with DODS using equally prepared GUV thus substantiating the DODS approach.

In order to perform experiments on a precisely studied system we monitored GUVs interacting with special substrates [SI6]. To prepare these, cleaned glass substrates were stamped with micro-scale grids of BSA-biotin which was further functionalized with neutravidin (NAV). The GUV membrane which exhibits biotin moieties, adheres to these grids but in-between the grids, the membrane continues to fluctuate (as shown in **Supplementary Fig. 8a and b**). This geometry ensures well defined measurement conditions without any lateral drift of the vesicles. Following reference [SI2], the DW-RICM data was theoretically corrected to account for the limited time resolution. Since both DW-RICM and DODS require dedicated microscopes, it was not possible to make one-to-one correspondence of the height traces. We therefore report the average and standard deviation of the fluctuation amplitude measured for 14 vesicles in each case. The two measurements were done successively on the same sample. The value extracted from DW-RICM data matches very well with DODS data.

Supplementary Note 4: Calculation of tension and dissipation from dACF

Accounting for system point spread function: The cross-section between the confocal volume and the membrane has a finite size, instead of being a single point $r = (x, y)$ on the membrane. In order to make quantitative comparison with theory, we account for blurring of the fluctuations by the point spread function $P(r)$ of the system

$$P(r) = \frac{2}{\pi\omega^2} \exp\left[-2\frac{r^2}{\omega^2}\right] \tag{7}$$

Here, ω is the radial radius of the laser beam at the axial position h_0 which increases with distance from the center of the point spread function (for definition of the coordinates see also **Fig. 2**) [SI1]

$$\omega^2 = \omega_0^2 \left(1 + \frac{\lambda^2 h_0^2}{\pi^2 n^2 \omega_0^4}\right) \tag{8}$$

with the refractive index n of the bulk medium and the wavelength λ of the excitation light. Measured fluctuations are thus a convolution of the real membrane fluctuations and $P(r)$. For the general principle, see Schmidt et al. [SI2] and references therein. Here, $P(r)$ is used to deconvolve the dACF which is fitted with a theoretical expression to calculate the membrane

bending rigidity κ, membrane tension σ (see following subsection). Note that if the dACF is not deconvoluted, both κ and σ are underestimated by about 20% (Authors, in preparation).

Tension and dissipation from fluctuations: In a model membrane, the fluctuations are determined by the membrane bending elasticity, κ, membrane tension, σ, and a dissipation depending on the bulk liquid viscosity, η. The theoretical description of the dynamics of model membranes is well known in the literature (for a review see Seifert [SI3] and references therein), which combined with the deconvolution above yields

$$\langle \delta h(\tau)\, \delta h(0) \rangle = \frac{k_B T}{2\pi} \int_{q_{\min}}^{\infty} dq \frac{1}{\kappa q^3 + \sigma q} \exp\left[-\frac{1}{4} q^2 \omega^2 - \frac{(\kappa q^3 + \sigma q)\tau}{4\eta} \right] \quad (9)$$

The integral runs over all modes that can be excited on the membrane, where the natural lower limit of the wave vector is given by the vesicle radius R and $q_{\min} = 1/R$. Note, that the Monge Gauge here assumes that on average the GUV membrane is flat (in fact, it is curved at large scale). Calculations in the quasi-spherical approximations yield values which can differ by up to 20% compared to an analysis performed in Monge Gauge. The systematic error can be reduced below 10% by choosing a slightly different lower cut off (Authors, in preparation). Here, for simplicity, we retain the flat membrane approximation and the conventionally used lower cut off. By using vesicles of typically 30 µm diameter and with a beam diameter of about 0.6 µm we ensured the validity of this approximation. For SOPC membrane, $\kappa = 20$ k_BT [6] is well known and was kept constant throughout the analyses. This strategy provides a way to sensitively determine σ and η. A typical fit is shown in **Fig. 3**. The corresponding tension is 0.23 µJ/m² and the viscosity is 1.15 mPas. As mentioned in the main text, this is in very good agreement with expectations. Note, however, that a refinement in the theoretical description (see [SI2] and Authors, in preparation), accounting for the viscosity of the inner buffer, which is $\eta_i = 1.4$ mPas in the presented system and different from the outer buffer PBS with $\eta_0 = 1.0$ mPas, can further improve the agreement between the estimated value and the DODS measurements.

Supplementary Note 5: Axial scans for intensity gradient control

DODS can be used to measure membrane fluctuations at variable distances away from the substrate spanning 0-100 µm. This is demonstrated here for measurements at the rim and centre of erythrocytes, close to the cell body and in the lamellipodium of macrophages, at the distal surface of GUV and in control measurements with SLBs. This spatial flexibility is due to the fact that DODS requires only axial intensity gradient information around the membrane average position. This gradient intensity is measured by axial scans across the membrane, which –for the objects in this study- returned Gaussian intensity distributions. Measurements are performed at the inflection point of the Gaussian, where the highest intensity gradient is reached. Moreover, any systematic distortion of the confocal volume, asymmetric fluorophore distribution, etc. will directly be visible through changes in the Gaussian intensity distribution. Thus, performing axial scans prior to each measurement yields high control over the actual intensity variation for height fluctuation conversion.

Supplementary References

[1] Palmer, A. G. & Thompson, N. L., Optical spatial intensity profiles for high order autocorrelation in fluorescence spectroscopy, *Appl. Opt.,* **28**, 1214-1220 (1989).
[2] Schmidt, D. et al., Signature of a nonharmonic potential as revealed from a consistent shape and fluctuation analysis of an adherent membrane, *Phys. Rev. X,* **4**, 021023 (2014).
[3] Seifert, U., Configurations of fluid membranes and vesicles, *Advances in Physics,* **46**, 13-137 (1997).
[4] Monzel, C., Fenz, S., Merkel, R. & Sengupta, K. Probing biomembrane dynamics by dualwavelength reflection interference contrast microscopy. *Chemphyschem* **10**, 2828–2838 (2009).
[5] Limozin L., Sengupta K., Quantitative reflection interference contrast microscopy (RICM) in soft matter and cell adhesion. *Chemphyschem*, **10**, 2752-2768, (2009)
[6] Monzel, C., Fenz, S. F., Giesen, M., Merkel, R. & Sengupta, K. Mapping fluctuations in biomembranes adhered to micropatterns. *Soft Matter* **8**, 6128-6138 (2012).
